# Transcriptomic signatures in peripheral CD4^+^T-lymphocytes may reflect melanoma staging and immunotherapy responsiveness prior to ICI initiation

**DOI:** 10.3389/fimmu.2025.1529707

**Published:** 2025-03-28

**Authors:** Eleni Palli, Matthieu Lavigne, Panagiotis Verginis, Themis Alissafi, Amalia Anastasopoulou, Georgios Lyrarakis, John M. Kirkwood, Helen Gogas, Dimitrios C. Ziogas

**Affiliations:** ^1^ First Department of Internal Medicine, Laikon General Hospital, National and Kapodistrian University of Athens - School of Medicine, Athens, Greece; ^2^ Institute of Molecular Biology and Biotechnology of the Foundation for Research and Technology - Biology Department, University of Crete, School of Medicine, Heraklion, Greece; ^3^ Laboratory of Biology, National and Kapodistrian University of Athens - School of Medicine, Athens, Greece; ^4^ Division of Hematology/Oncology, University of Pittsburgh, School of Medicine, Pittsburgh, PA, United States

**Keywords:** immune checkpoint inhibitors, immunotherapy, resistance, CD4+T-cells, differentially expressed genes, bulk RNA-seq

## Abstract

**Background and purpose:**

Promoting adaptive immunity with ICIs has drastically improved melanoma prognosis, but not for all patients. Some cases relapse in the first few months, while others keep durable benefit, even after immunotherapy discontinuation. To identify cellular/molecular signatures in peripheral blood that could differentiate advanced from metastatic melanoma and predict dynamics for primary/secondary immune escape, we examined 100 consecutive patients with stage III/IV melanoma scheduled to start ICIs.

**Materials and methods:**

At melanoma diagnosis, a multiparameter flow cytometric analysis and purification scheme using standard conjugated antibodies were performed for all individuals prior to ICI initiation. In each stage(III/IV) according to their RFS/PFS, we retrospectively selected the cases with the clearest clinical outcomes and focused our analysis on the extreme responders(n=7) and non-responders(n=7) to characterize the transcriptomes of circulating CD4^+^T-cells by bulk RNA-seq, Differential Expression Analysis(DEA)and Gene Ontology(GO)enrichment analysis. Based on our selected patient cohort, we examined for differentially expressed genes(DEGs)and key-pathways that appear preferentially activated in stage III vs. IV melanoma, and in long vs. short immunotherapy responders.

**Results:**

Although circulating immune-cells did not numerically differ in both sets of analysis(staging and ICI responsiveness), DEA and GO data showed that patients could be clustered separately, identifying 189vs.92 DEGs in stage IV/III and 101vs.47 DEGs in early progressors/long responders. These DEGs were functionally implicated in distinct pathways. For metastatic cases: inflammatory response(logp-value=-9.2:ADGRE5/2,CYBA,GRN,HMOX1,IRF5,ITGAM), adaptive immunity(logp-value=-7.7:CD1C,CD74,CYBB,NCF2,CTSA,S100A8/9,BCL3,FCER1G), T-cell activation(logp-value=-6.3:BCL3,CD1C,CD74,FCER1G,FGL2)and lipid metabolism/catabolism(logp-value=-2.5/-2.6:ARF3,GPX1,MVD,OCRL,PCCB,CTSA,PNPLA2,NAGLU,GBA2,ABHD4); while in early-progressors to ICIs: immune effector processing(logp-value=-13.7:BCL6,FGR,HLA-DQA1/DQB1,HLA-DRA,HLA-DRB1/DRB5,NKG7,SLC11A1,TYROBP,SPON2,HAVCR2),PD-1(logp-value=-10.2:HLA-DQA1/DQB1,HLA-DRA,HLA-DRB1/DRB5)and IFN signaling(logp-value=-8.5: HLA-DQA1/DQB1,HLA-DRA,HLA-DRB1/DRB5,NCAM1,IFITM3),positive regulation of T-cell activation(logp-value=-7.7:BCL6,HLA-DQA1/DQB1,HLA-DRA,HLA-DRB1/DRB5,SASH3,HAVCR2)and CD28 co-stimulation(logp-value=-10.3:HLA-DQA1/DQB1,HLA-DRA,HLA-DRB1/DRB5), supporting an immune-mediated behavior.

**Conclusions:**

Specific pathways and marker genes in the peripheral CD4^+^T-cells may predetermine melanoma staging and immunotherapy resistance.

## 1 Introduction

Melanoma, even from its less advanced stages, has a natural propensity to spread and metastasize that is critically regulated via a constant interaction with the immune system. Blocking melanoma escape via inhibiting checkpoint molecules has remarkably improved the clinical outcomes of patients with advanced or metastatic disease. In the adjuvant setting, more than half of melanoma patients treated with anti-PD-1 agents survive free of their disease after 5 years (5-year Relapse-Free Survival, RFS% for pembrolizumab: 55.4% and for nivolumab: 51.7%) (1, 2), while in the metastatic setting, the 10-year overall survival rate (OS%) reached to 34% for pembrolizumab (3), to 37% for nivolumab and to 43% for nivolumab and ipilimumab combination in treatment-naive cases (4). However, not all melanoma patients have a standard evolution pattern and a similar response to immune checkpoint inhibitors (ICIs). Some of them exhibit an early relapse or progression in the first few months with no clinical benefit, while others experience durable disease control and prolonged survival, even after the discontinuation of immunotherapy (5). It is not clear how melanoma cells overcome immunosurveillance and enter into the circulation and whether any cellular or molecular signatures in tumor or blood cells could predict success or lack of response (primary resistance) to ICIs (5).

Until now, the majority of biomarker analyses have focused on the tumor cells per se, the tumor-infiltrating lymphocytes (TILs) and the surrounding tumor microenvironment (TME) to identify specific intra-tumoral features that could predetermine the melanoma behavior (e.g., plasticity and migratory potential) and the outcomes to anti-PD-1 therapy (6). An aspect that has received less attention is that the dynamic cross-talk of melanoma and immune system initiates from TME, but extends also outside the TME, and involves many different peripheral immune cells (e.g., T-cells (7–9), NK cells (10) and B-cells (11)) (12). Among implicated immune subpopulations, T-cells are the second most frequently detected subpopulation in human tumors after macrophages, and are extensively studied in diverse cancer types (13–17). They can polarize immune responses as T-helper cells (CD4^+^cells); orchestrate humoral reactions as T-follicular helper cells (a specialized subset of CD4^+^ cells); modulate the activity of effector cells as T_reg_ cells (there are both CD4^+^ and CD8^+^T_reg_ cells); or directly kill targeted cancer cells as cytotoxic T-cells (mainly CD8^+^ and a small subset of CD4^+^) (18). During the early melanoma stages, naïve T-cells will be primed in the draining lymph nodes, followed by their concomitant activation and migration to the TME (12, 19). Recent studies have further highlighted the multiple involvement of peripheral CD4^+^T-cells in both innate and adaptive immune response (20–22), enhancing the antigen presentation machinery, increasing CD8^+^T-cell effector differentiation (21, 23), expressing even direct cytotoxic activity (24), driving B-cell activation and antibody affinity maturation (20–22), sustaining the immune surveillance during melanoma evolution via targetable checkpoint molecules (20–22, 25). For all these abovementioned reasons, the CD4^+^ subpopulation was predetermined in our study as a potential cellular biomarker to explore its distinct transcriptomic profiles.

The difficulty in obtaining clinically useful cellular or molecular biomarkers from tissue or blood reflects the deep complexity and variability of melanoma biology. Particularly, the peripheral blood offers an accessible and minimally invasive “biopsy” that could help to identify patients at higher risk of progression or relapse, as well as those most likely to benefit from an ICI. Despite the extensive research, data on blood-borne biomarkers correlating with clinical outcomes are limited; myeloid-derived suppressor cells (MDSCs) have been found to contribute in melanoma evasion, being also negatively associated with the natural course of several other malignancies (7, 26), while absolute lymphocyte count and serum LDH are characterized by higher prognostic rather predictive value of response to ICIs (27–30), as well as by low specificity. Recently, bioinformatics technology and high-throughput sequencing platforms gave the opportunity for a deeper molecular analysis of diverse circulating immune subpopulations in order to reveal melanoma-related genes with significant differences in expression (differentially expressed genes, DEGs) in these cellular subsets that can be used as blood-borne biomarkers and to detect potential targetable biological processes for further functional research (31).

In our cohort, a multiparameter flow cytometry approach was used to detect numerical differences in the main circulating immune cellular subpopulations between patients with locally advanced (stage III) or metastatic (stage IV) melanoma who were scheduled to start ICIs. Next, peripheral CD4^+^T-cells were isolated from retrospectively selected patients according to their RFS/PFS, focusing on those with the best and worst behavior to ICIs in each stage. Bulk RNA sequencing followed by differential gene expression analysis (DEA) and gene ontology (GO) enrichment analysis were performed in the peripheral CD4^+^T-cells from the patients with the clearest clinical outcomes (extreme responders or non-responders) to limit the potential technical difficulties to deconvolute multi-parameter and difficult to interpret signatures. Doing this, we tried to extract more specific prognostic/predictive gene sets associated with metastatic spread and responsiveness to ICIs (32). While being a hypothesis-generating study, the aim of this work is to show that there is a well-defined segregation of specific transcriptional patterns that can be detected in circulating CD4^+^T-cells among patients with different staging; and these transcriptomic findings may serve as stepping stones to future studies that will further elucidate the immune-mediated melanoma behavior and will drive to more personalized immunotherapy decisions.

## 2 Materials and methods

### 2.1 Study design

This is an exploratory, hypothesis-generating, study designed to identify specific DEGs and GO enriched pathways from peripheral blood CD4^+^T-cells collected at the time of initial patient diagnosis and propose candidate genes that can predict responsiveness to immunotherapy.

### 2.2 Human subjects-assessment of response

The study population comprised 100 consecutive newly diagnosed patients with advanced or metastatic melanoma at our university-affiliated center (Laikon General Hospital, School of Medicine, National and Kapodistrian University Athens, Greece) between February 2019 and August 2019. The study follow-up ended in August 2022. All included patients were adults (≥18years of age at diagnosis), had a recent histologically confirmed diagnosis of stage III or stage IV melanoma according to the latest version of the International Staging System (AJCC, 8th Edition) and were about to start the following ICIs (either as adjuvant or first line treatment): Nivolumab (anti-PD-1, Opdivo: 240mg flat dose IV every 2 weeks), Pembrolizumab (anti-PD-1, Keytruda: 200mg flat dose IV, every 3 weeks), or Nivolumab/Ipilimumab (1mg/kg Nivolumab IV and subsequently 3mg/kg Ipilimumab IV for 4 times within a 3-week interval; combination therapy was followed by Nivolumab maintenance). Prior initiating their immunotherapy, all patients underwent complete clinical examination and peripheral blood sampling. Informed written consent was obtained from every participant before the collection of blood samples. The study protocol was approved by the local Ethics Committee and all procedures in this study involving human subjects were performed in accordance with the Internal Review Board of our institution as well as with the Declaration of Helsinki (1964) and its later amendments. Tumor response was assessed by using Response Evaluation Criteria in Solid Tumors (RECIST), version 1.1, every 8-12 weeks. Retrospectively, we re-evaluated our cohort of ICI-treated melanoma patients and split them to early progressors (e.g., RFS/PFS<12 months) and to long responders (e.g., RFS/PFS>12 months). For the subsequent Differential Expression Analysis and Gene Ontology enrichment analysis, we exclusively focused on the 7 cases that showed the best clinical outcomes (e.g., the 7 cases with the longest RFS/PFS) and the 7 extreme non-responders to ICIs (e.g., the 7 cases with the shortest RFS/PFS) in each stage, according to their RFS/PFS.

### 2.3 Blood sample collection

Peripheral blood (5-7 mL) was collected in EDTA Vacutainer blood collection tubes (Becton Dickinson) from melanoma subjects. Peripheral blood mononuclear cells (PBMC) were isolated on Histopaque-1077 density gradient (cat. #10771, Sigma), according to the manufacturer’s instructions. Blood was initially diluted 1:1 with PBS and carefully layered over Histopaque medium. Ficoll gradients were centrifuged at 1800 rpm for 30 minutes with no brake at room temperature. After the centrifugation, the PBMC layer was collected from each sample and cells were washed with PBS.

### 2.4 Flow Cytometry immunophenotyping and gating strategy

The PBMCs pellet was resuspended in 5% FBS/PBS followed by immunophenotypic analysis. MFI (mean fluorescence intensity) values were not normalized to a Fluorescence Minus One (FMO) control but using a single-cell staining control. Before any other action, the staining protocol was standardized for characterizing myeloid and T-cell immune cell subpopulations. Immunostaining was done using an unstained sample (a sample with the staining buffer but without the addition of antibodies to remove the background and debris), single-cell staining samples (containing each different antibody in a different separate sample), and a sample with all antibodies of interest. The **Supplementary Table 1** describes the antibodies used in Flow Cytometry. The levels of compensation were determined by using the single stain controls as they provide the requisite information to identify the amount of spillover of each antibody into a non-specific binding context and correct for this spillover through the process of compensation. PBMCs were stained with carefully selected conjugated antibodies in order to characterize and identify specific immune cell subtypes, including three monocytes’ subpopulations from myeloid lineage (HLA-DR^+^CD14^+^CD16^-^, HLA-DR^+^CD14^+^CD16^+^ and HLA-DR^+^CD14^-^CD16^+^) and MDSCs (HLA-DR^-^CD33^+^CD15^+^) and CD4^+^T-cells (helper T-cells), CD8^+^T-cells (cytotoxicT-cells), and CD4^+^FOXP3^+^T-cells (Tregs) from lymphoid lineage. Intracellular staining was performed using eBioscience™ Foxp3/Transcription Factor staining buffer set (#00-55-23-00) according to the manufacturer’s instructions. Cells of interest were acquired on a FACS ARIA III (BD Biosciences) using BD FACSDIVA v8.0.1 software (BD FACSDiva Software, RRID: SCR_001456). Flow cytometry analysis was performed with FlowJo software and MFI was assessed by using the algorithm in the FlowJo software (FlowJo, RRID: SCR_008520). The gating strategy is presented in **Supplementary Figure 1** (**Supplementary Figures 1A-C** **).** The MFI measurement was calculated by the sum of the integrated density (fluorescence intensity of cells) divided by the total number of each cell subpopulation. Based on the absolute number of MFI, the expression levels of checkpoint molecules PD-1 for T-cells as well as PD-L1 and PD-L2 for myeloid cells were also estimated.

### 2.5 Fluorescent-activated cell sorting of CD4^+^T-cell subpopulation

After PBMCs isolation, an amount of approximately 10x10^6^ cells per sample was resuspended in 1ml of freezing medium and was stored in cryovials at -80°C. In order to sort CD3^+^CD4^+^cell subpopulation from frozen PBMCs, thawing procedure was followed for the selected 28 samples. Briefly, cryopreserved PBMCs were resuspended in a nutrient medium consisting of FBS and plain RPMI (Thermo Fisher Scientific, USA) and placed into 37°C on water bath for a few seconds. Centrifugation was followed at 400g for 7 minutes at room temperature and the pellet was resuspended in 5% FBS/PBS. PBMCs were stained with the conjugated antibodies CD3 (PE, Cat. #300308, Clone: HIT3a, Biolegend) and CD4 (PerCP/Cy5.5, Cat. #317428, Clone: OKT4, Biolegend) to identify and sort CD3^+^CD4^+^T-lymphocytes. After sorting, the CD3^+^CD4^+^T-cell population was resuspended in β-mercaptoethanol and cell lysis solution, to continue with RNA extraction.

### 2.6 Isolation of RNA and 3′ RNA sequencing

Total RNA was extracted using NucleoSpin RNA XS (Macherey-Nagel; cat. #740955.50) as per the manufacturer’s protocol. The quantity and quality of RNA samples were analyzed using Agilent RNA 6000 Nano kit with the bioanalyzer from Agilent. The 26 of 28 RNA samples with RNA integrity number (RIN)>7 were used for library construction using the 3′ mRNA-Seq Library Prep Kit FWD for Illumina (QuantSeq-LEXOGEN) as per the manufacturer’s instructions. Amplification was controlled for obtaining optimal unbiased libraries across samples by assessing the number of cycles (ranging from 20-24) required by qPCR. Indexes were used as described in **Supplementary Table 2**. DNA High Sensitivity Kit for bioanalyzer was used to assess the quantity and quality of libraries, according to the manufacturer’s instructions (Agilent). Libraries were multiplexed and sequenced on an Illumina Nextseq 500 at the genomics facility of IMBB FORTH according to the manufacturer’s instructions. After QC we discarded two samples that did not show satisfactory sequencing metrics (M11 and M96). For the final samples used in the analysis, we show the number of reads obtained in the **Supplementary Table 2**.

### 2.7 Differential expression analysis and gene ontology enrichment analysis of bulk RNA sequencing data

The quality of the raw sequences in output FASTQ files was assessed with the FastQC software (33). Reads were aligned to the human (hg38) genome (34) with the Hisat2 aligner (35) (hisat2 -p32 -x $REFERENCE_GENOME -q fastq/$FILE_ID.fastq -S $FILE_ID.sam –score-min L,0,-0.5 -k 2). Htseq-counts (36) was utilized to summarize reads at the gene level (htseq-count -f bam -s yes-I gene_id bam/$FILE_ID.bam data/refs/Homo_sapiens/UCSC/hg38/Annotation/Genes/genes.gtf>$COUNTS_DIR/NGS$FILE_ID). DEA was conducted by running EdgeR (37) via SARTools 1.5.0 (38) using weighted trimmed mean of the log expression ratios (trimmed mean of M values (TMM)) as a. normalization method, no batch correction and a cpmCutoff of 1 for genes to be tested. For each pairwise comparison (stage IV, S4 vs. stage III, S3) and early progressors (Short RFS/PFS, S) vs. long responders (Long RFS/PFS, L), DEGs in either group of the comparison were defined by applying the following thresholds |Log2FC or LFC| >1 and *p*-value <0.05, which was considered statistically significant in order to limit the stringency of FDR testing and help us to account for anticipated interpatient variability. Heatmaps and boxplots were created in R with an in-house-developed script (available upon request) relying on the complex heatmap R package. GO analysis and Transcription Factor Binding Sites (TFBS) enrichment analysis were run on the web tool Metascape (39).

## 3 Results

### 3.1 Patients groups

The entire cohort of 100 consecutive melanoma patients included 57 cases with stage III and 43 with stage IV disease (with equal gender distribution, 51% males in both stage groups). At the time of diagnosis, the median age of included patients with melanoma stage III and IV were 67.51 and 66.77 years, respectively. Both stage groups had similar proportions of patients with elevated LDH at melanoma diagnosis (LDH>UNL, 25% and 37%, p=0.192; median LDH: 230IU and 239IU, p=0.635, respectively). The only significant differences were detected in the therapeutic approach, where the majority of patients with stage III disease received monotherapy with nivolumab following the physicians’ decision (60%, compared to 28% of individuals with melanoma stage IV) in parallel with the reasonably limited use of nivolumab/ipilimumab to only 3 patients (5%) in the adjuvant setting, while in the metastatic setting 39% of cases received the ICI-doublet (p<0.0001). Despite these differences in treatment selection, median RFS was not reached in ICI-treated patients with melanoma stage III and median PFS was 21.6 months for ICI-treated patients with melanoma stage IV (log-rank test, p=0.017) while median OS was not reached for both stage groups, probably due to immature data (log-rank test, p=0.485).

After retrospectively re-grouping our 100 patients with advanced and metastatic melanoma according to their RFS/PFS, 32 cases relapsed/progressed under immunotherapy in less than 12 months (“early progressors”) while 68 had no event of progression or death in the first 12 months of immunotherapy (“long responders”) (median RFS/PFS: 4.4 months in early progressors vs. not reached in long responders, log-rank test p<0.0001). Gender and age distributions were comparable to both groups. No differences were found among the administered ICI-regimens in this set of analysis. As expected, the values of LDH (median LDH: 242IU vs. 229.5IU, p=0.010) and the proportions of individuals with LDH>UNL (44% vs. 24%, p=0.005) were significantly higher in the early progressors compared to the long responders. In consistence with other melanoma studies (40), the RFS/PFS was a surrogate marker of OS (median OS 14.3 in early progressors vs. not reached in long responders, log-rank test p<0.0001). Baseline demographic characteristics and outcomes of included patients for both sets of analysis are presented in **Table 1**.

**Table 1:** Patient and melanoma characteristics at the time of blood collection and immunotherapy outcomes, according to their initial staging groups and according to their retrospective stratification based on their RFS/PFS to ICIs.

Baseline characteristics	Total (n=100)	Stage III (n=57)	Stage IV (n=43)	p-value (St III vs. IV)	Early Progressors (n=32)	Long Responders (n=68)	p-value (Early Progressors vs. Long Responders)
Male gender, (%)	51 (51)	29 (51)	22 (51)	0.981	18 (56)	33 (48)	0.524
Median age at melanoma diagnosis, range (years)	67.4 (18.8-91.5)	67.5 (18.8-91.3)	66.8 (33.2-91.5)	0.168	65.5 (24.2-91.5)	67.9 (18.8-89.8)	>0.999
Median LDH at melanoma diagnosis, (IU)	235.5	230.0	239.0	0.635	242.0	229.5	**0.010**
LDH>UNL, (%)	30 (30)	14 (25)	16 (37)	0.192	14 (44)	16 (24)	**0.005**
Nivolumab, (%)	46 (46)	34 (60)	12 (28)	**0.002**	15 (49)	31 (46)	>0.999
Pembrolizumab, (%)	34 (34)	20 (35)	14 (33)	0.834	12 (38)	22 (32)	0.655
Nivolumab/Ipilimumab, (%)	20 (20)	3 (5)	17 (39)	**<0.0001**	8 (25)	12 (18)	0.428
RFS/PFS from blood collection, median (months)	Not reached	Not reached	21.6	**0.017** log-rank test	4.4	Undefined	**<0.0001** log-rank test
OS from blood collection, median (months)	Not reached	Not reached	Not reached	0.485 log-rank test	14.3	Undefined	**<0.0001** log-rank test

LDH, Lactate Dehydrogenase; RFS, Relapse Free Survival; Progression Free Survival; OS, Overall Survival.

Bold values are reaching to statistical significance.

### 3.2 Immunophenotyping profile of peripheral blood immune cells in melanoma patients according to their stage and their RFS/PFS

**Figure 1:**
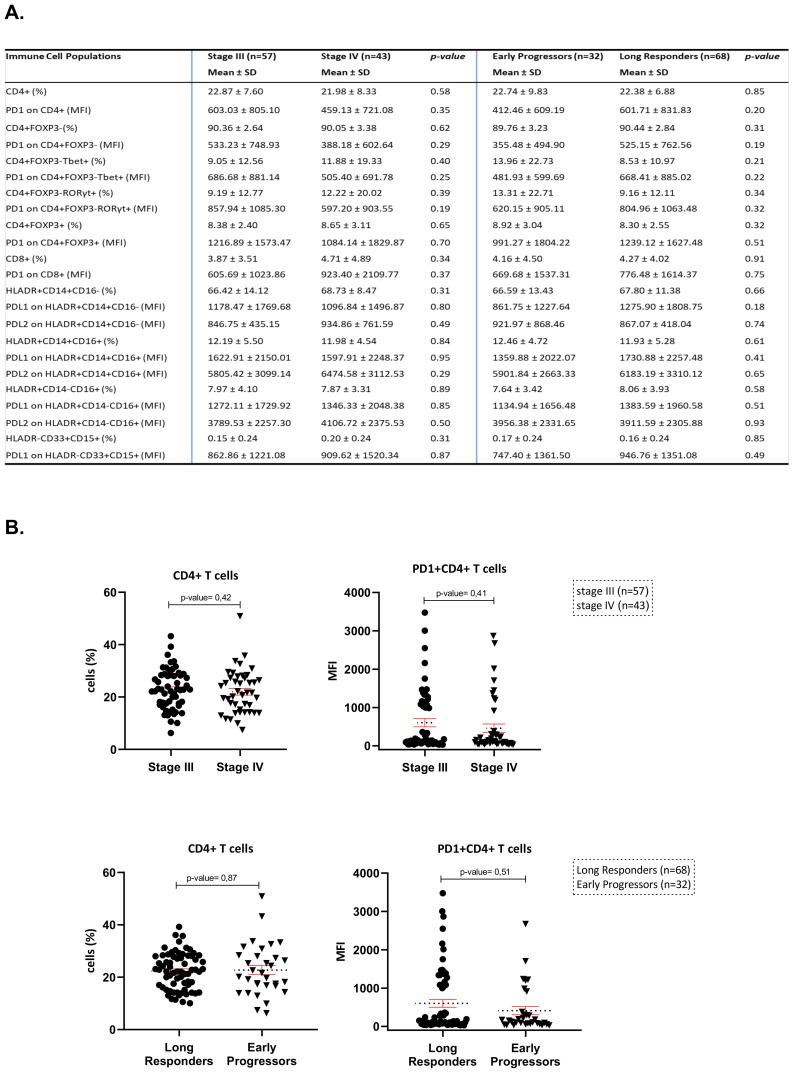
**(A)** Flow cytometry results of the main immune cell subpopulations of the peripheral blood in both sets of analysis (stage III vs. stage IV and long responders vs. early progressors). **(B)** Scatter plots of CD4^+^subpopulations and PD-1^+^CD4^+^subpopulations in both sets of analysis (stage III vs. stage IV and long responders vs. early progressors).

The flow cytometry analysis (FCA) of immune cell subpopulations between stage III (n=57) and stage IV (n=43) melanoma patients did not reveal any statistically significant differences. More specifically, the percentages of CD4^+^, CD4^+^FOXP3^-^, CD4^+^FOXP3^+^ and CD8^+^T-cells (mean ± SD: 22.87 ± 7.60 vs. 21.98 ± 8.33, p=0.58; 90.36 ± 2.64 vs. 90.05 ± 3.38, p=0.62; 8.38 ± 2.40 vs. 8.65 ± 3.11, p=0.65 and 3.87 ± 3.51 vs. 4.71 ± 4.89, p=0.34, respectively) as well as the percentages of monocytes’ subtypes, HLADR^+^CD14^+^CD16^-^, HLADR^+^CD14^+^CD16^+^, HLADR^+^CD14^-^CD16^+^, and HLADR^-^CD33^+^CD15^+^ myeloid cells (mean ± SD: 66.42 ± 14.12 vs. 68.73 ± 8.47, p=0.31; 12.19 ± 5.50 vs. 11.98 ± 4.54, p=0.84; 7.97 ± 4.10 vs. 7.87 ± 3.31, p=0.89 and 0.15 ± 0.24 vs. 0.20 ± 0.24, p=0.31, respectively) were comparably similar between the two groups by stage (**Figure 1A**). Moreover, the MFI of PD-1 on CD4^+^, CD4^+^FOXP3^+^, CD8^+^T-cells (mean ± SD: 603.03 ± 805.10 vs. 459.13 ± 721.08, p=0.35; 1216.89 ± 1573.47 vs. 1084.14 ± 1829.87, p=0.7 and 605.69 ± 1023.86 vs. 923.40 ± 2109.77, p=0.37, respectively) have shown no significant differences between stage III and stage IV melanoma patients. In the same concept, we have analyzed PD-L1 and PD-L2 on HLADR^+^CD14^+^CD16^-^ monocytes (mean ± SD: 1178.47 ± 1769.68 vs. 1096.84 ± 1496.87, p=0.8; 846.75 ± 435.15 vs 934.86 ± 761.59, p=0.49), on HLADR, HLA-DR^+^CD14^+^CD16^-^, HLA-DR^+^CD14^+^CD16^+^ and HLA-DR^+^CD14^-^CD16^+^, as well as the HLA-DR^-^CD33^+^CD15^+^ myeloid cells, we have not found any statistically significant differences (mean ± SD, 66.59 ± 13.43 vs. 67.80 ± 11.38, p=0.066; 12.46 ± 4.72 vs. 11.93 ± 5.28, p=0.61; 7.64 ± 3.42 vs. 8.06 ± 3.93, p=0.58 and 0.17± 0.24 vs. 0.16 ± 0.24, p=0.85, respectively) between the two groups of stage III and IV (**Figure 1A**).

Moving to the other set of analysis between early progressors (n=32) and long responders (n=68), the percentages of lymphoid and myeloid subpopulations were similar (**Figure 1A**). In addition, the MFI of immune checkpoint molecules of PD-1 on CD4^+^, on CD4^+^FOXP3^+^, and on CD8^+^T-cells have shown no significant differences (mean ± SD: 412.46 ± 609.19 vs. 601.71 ± 831.83, p=0.2; 991.27 ± 1804.22 vs. 1239.12 ± 1627.48, p=0.51 and 669.68 ± 1537.31 vs. 776.48 ± 1614.37, p=0.75, respectively) and of PD-L1 and PD-L2 on HLA-DR^+^CD14^+^CD16^-^monocytes (mean ± SD, 861.75 ± 1227.64 vs. 1275.90 ± 1808.75, p=0.18; 921.97 ± 868.46 vs. 867.07 ± 418.04, p=0.74), on HLADR^+^CD14^+^CD16^+^monocytes (mean ± SD: 1359.88 ± 2022.07 vs. 1730.88 ± 2257.48, p=0.41; 5901.84 ± 2663.33 vs. 6183.19 ± 3310.12, p=0.65), on HLADR^+^CD14^-^CD16^+^monocytes (mean ± SD: 1134.94 ± 1656.48 vs. 1383.59 ± 1960.58, p=0.51; 3956.38 ± 2331.65 vs. 3911.59 ± 2305.88, p=0.93) and PD-L1 on HLA-DR^-^CD33^+^CD15^+^myeloid cells (mean ± SD, 747.40 ± 1361.50 vs. 946.76 ± 1351.08, p=0.49) did not also differ significantly. All immunophenotyping profiles of FCA results between the two sets of analysis are presented in **Figure 1**. Given that CD4^+^T-cells was the most abundant subpopulation in both sets of FCA and its role in adaptive immunity, this specific subpopulation entered into the focus for subsequent study. The scatter plot of **Figure 1B** depicts the similar percentages and MFI of peripheral CD4^+^ and PD-1^+^CD4^+^T-cells between patients with stages III and IV and between early progressors and long responders.

### 3.3 Transcriptomic signatures of peripheral CD4^+^T-cells in patients with stage III and IV melanoma

**Figure 2:**
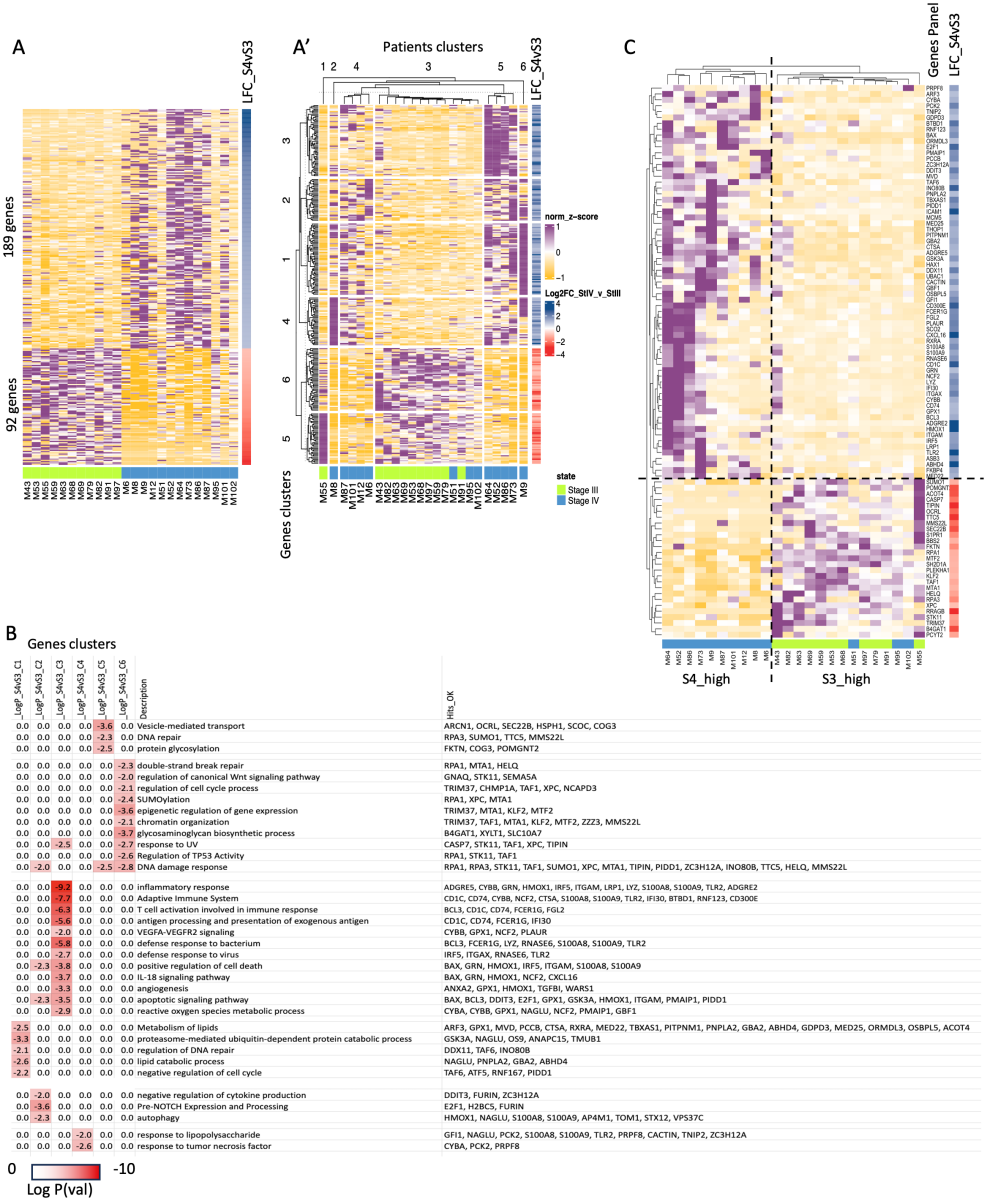
Transcriptomic analysis of CD4^+^T-cells from peripheral blood prior to ICI initiation of patients with melanoma stage III and IV. **A.**The panel A gives a general overview of normalized expression (Z-score) of unclustered, deregulated genes (rows) that are sorted according to LFC (shown in a separate column, most up to most down) and selected patients are grouped arbitrarily according to their stage III and stage IV melanoma diagnosis (92 upregulated genes in patients with stage III and 189 upregulated genes in stage IV melanoma) (columns, see Methods). Column labels are derived from sampling ID. **A’.**The panel A’ is k-mean unsupervised clustering of patients (columns) and genes (rows) according to normalized expression values (Z-score) to identify group of genes that characterize specific patients. **(B)** Gene Ontology (GO) analysis using the cluster of genes found in the panel A’ for stage III and stage IV. Darker red color signifies most enriched (decreasing -Log Pval) GO categories and gene names contributing to the signatures are highlighted on the right. **(C)** Same as in the panel A’, the panel C is a manually curated subset of the gene hits from GO analysis focusing on particular functions shown in **(B)** Labeling for samples: e.g., S4L means a patient with stage IV melanoma and long PFS under while S3S means a patient with stage III melanoma and short RFS under immunotherapy experiencing an early relapse. LFC: the Log2 Fold Change of the gene in the one group compared to the other group (control group). Positive LFC values indicate upregulation relative to control, while negative LFC values indicate downregulation.

RNA-seq followed by DEA and GO enrichment analysis was performed to discover specific signatures of peripheral CD4^+^T-cells (e.g., gene functions and pathways relationships) prior to immunotherapy initiation. To avoid anticipated difficulties in analysis of patients with heterogenous clinical outcomes, we focused on the cases that showed most extreme clinical outcomes, including: the 7 best (longest RFS/PFS) and the 7 worst responders to ICIs (shortest RFS/PFS) in each stage (III and IV, total 28 samples) retrospectively (see Methods). The RNA isolation procedure was assessed for total RNA degradation prior to proceeding to library preparation and sequencing. Poor RNA quality (2 samples) and low metrics in RNA-seq fastQC (2 samples)(see Methods) led us to exclude 4 out of our selected 28 samples (1 in the metastatic subgroup and 3 in the adjuvant subgroup). We thus used a still representative number of 24 samples to compare the gene expression levels between stage III and IV melanoma patients. We identified 189 upregulated genes in patients with stage IV (**Figure 2A**) and in parallel, these genes showed lower expression in stage III melanoma cases. In contrast, we found 92 upregulated genes in patients with stage III melanoma that were downregulated in individuals with stage IV melanoma (**Figure 2A**). The identified DEGs defined a robust clustering of the diagnosed patients (**Figure 2A’**, except from 3 cases with stage IV melanoma, M51, M95 and M102, resembling stage III patterning in patient cluster 3) and constitute an exploitable signature for molecular staging. In particular, we found segregation of stage IV patients in clusters 2 + 4 and 5 + 6, while stage III patients clustered mainly in 1 + 3 patients’ clusters. As a consequence, we noted that genes upregulated in stage IV were functionally implicated in distinct relevant GO categories such as inflammatory response (log p-value=-9.2: ADGRE5/2, CYBA, GRN, HMOX1, IRF5, ITGAM, etc.), adaptive immune system’s processes (log p-value=-7.7: CD1C, CD74, CYBB, NCF2, CTSA, S100A8/9, BCL3, FCER1G, etc.), T-cell activation in immune response (log p-value=-6.3: BCL3, CD1C, CD74, FCER1G, FGL2, etc.) in gene cluster 3, lipids’ metabolism (log p-value=-2.5: ARF3, GPX1, MVD, OCRL, PCCB, CTSA, PNPLA2, etc.), lipid catabolic process (log p-value=-2.6: NAGLU, PNPLA2, GBA2, ABHD4, etc.) and proteasome-mediated ubiquitin-dependent protein catabolic process (log p-value=-3.3: GSK3A, NAGLU, OS9, ANAPC15, TMUB1, etc.) in gene cluster 1 (**Figure 2B**, **Supplementary Table 2B**). In patients with stage III, GO pathways of cellular response to DNA damage response (log p-value=-2.8: RPA1/3, STK11, SUMO1, XPC,TIPIN, etc.), response to ultraviolet (UV) damage (log p-value=-2.7: CASP7, STK11, TAF1, XPC, TIPIN, etc.), double-strand break (DSB)(log p-value=-2.3: RPA1, MTA1, HELQ, etc.), protein glycosylation (log p-value=-2.5: FKTN, COG3, POMGNT2, etc.) and vesicle-mediated transport (log p-value=-3.56: ARCN1, OCRL, SEC22B, HSPH1, SCOC, COG3, etc.) were more enriched. A curation of particularly relevant genes is presented in **Figure 2C** and shows how they could constitute a relatively robust panel of genes that control functions involved in melanoma progression and that can be used to segregate patients with advanced melanoma from those with metastatic disease (despite the few exceptions). Therefore, harvesting RNA from CD4^+^T-cells and performing RT-qPCR on these DEGs, we are able to pre-determine the advanced or metastatic status of melanoma. This is the first report suggesting that melanoma staging may be detectable by analysis of the peripheral CD4^+^T-cell profiles.

### 3.4 Transcriptomic signatures of peripheral CD4^+^T-cells in long responders and early progressors to ICIs

**Figure 3:**
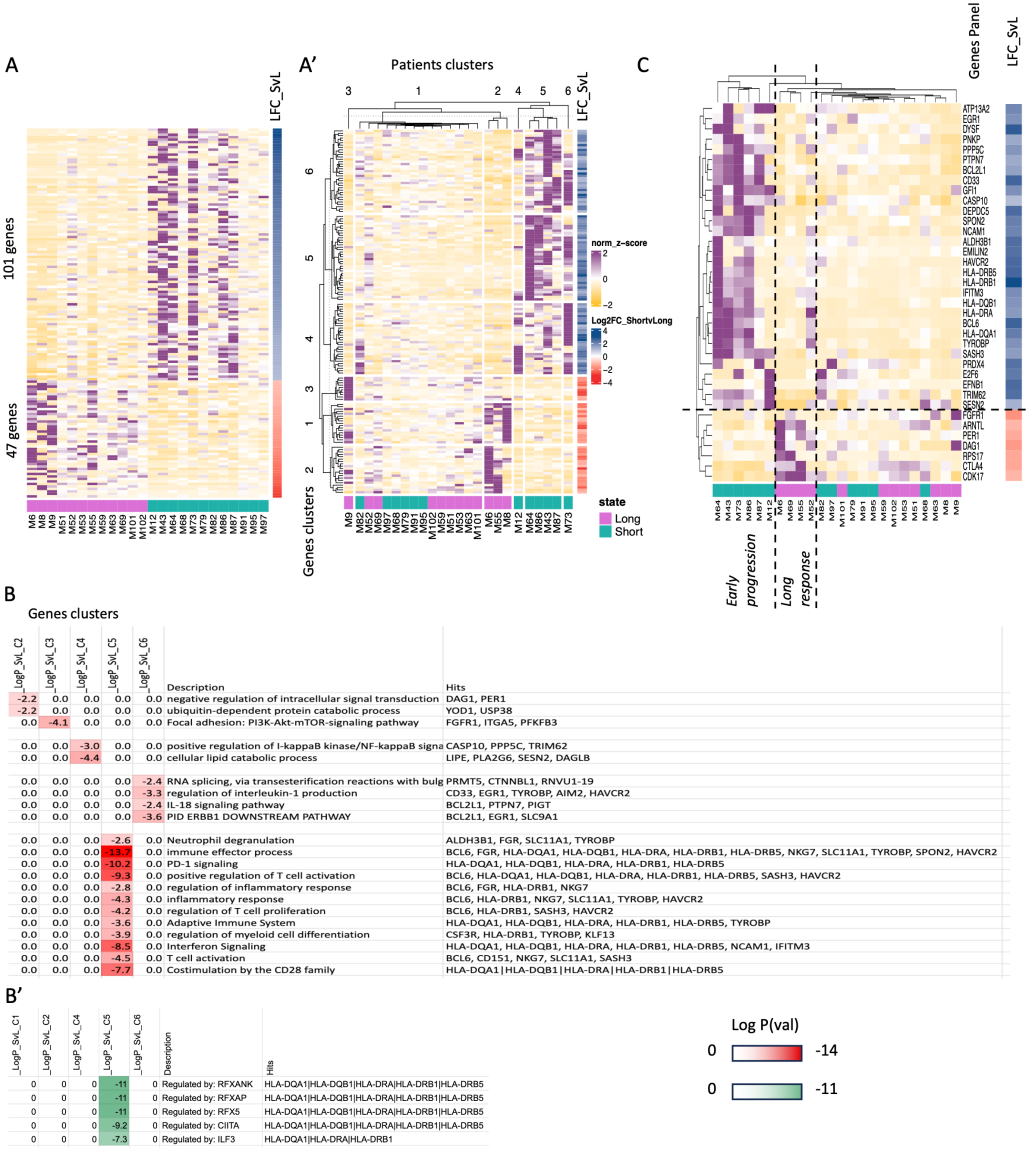
Transcriptomic analysis of CD4^+^T-cells from peripheral blood prior to ICI initiation of long responders and early progressors to immunotherapy. **(A)** and A’Heatmap showing normalized expression (Z-score) levels (rows) for DEGs between the selected patients with extreme clinical outcomes stratified them as long responders and early progressors under ICIs. (columns, see Methods). The panel A gives a general overview of normalized expression (Z-score) of unclustered, deregulated genes (rows) that are sorted according to LFC (shown in a separate column, most up to most down) and selected patients are grouped arbitrarily according to their immunotherapy response (47 upregulated genes in long responders and 101 upregulated genes in early progressors)(columns, see Methods). Column labels are derived from sampling ID. The panel A’ is k-mean unsupervised clustering of patients (columns) and genes (rows) according to normalized expression values (Z-score) to identify group of genes that characterize specific patients. **(B)**. Gene Ontology (GO) enrichment analysis using the clusters of genes found in A’ for long responders and early progressors under ICIs. Darker red color signifies most enriched GO categories and gene names contributing to the signatures are highlighted on the right. B’. Transcription Factor Binding Sites (TFBS) enrichment analysis found on the promoter of genes found in A’. (darker green signifies most enriched TFBS categories and gene names contributing to the signatures are highlighted on the right. **(C)**. Same as in panel A’, the panel C is a manually curated subset of the gene hits from GO analysis focusing on particular functions shown in B. LFC: the Log2 Fold Change of the gene in the one group compared to the other group (control group). Positive LFC values indicate upregulation relative to control, while negative LFC values indicate downregulation.

Looking for additional ICI-specific transcriptomic signatures, a similar analysis of CD4^+^T-cells in the same selected group of extreme individuals with advanced/metastatic melanoma (n=24) was performed to recognize candidates with greater likelihood for durable response or early relapse/progression upon immunotherapy. We first identified DEGs between long responders (LONG, n=12) and early progressors (SHORT, n=12) with either III or IV stages (**Figure 3A**) and present in the heatmap i) 101 genes found to be upregulated in early progressors (and down-regulated in long responders) and ii) 47 genes upregulated genes in long responders (and down-regulated in early progressors). Importantly, these DEGs defined a relatively robust clustering of a fraction of the diagnosed patients (**Figure 3A’**, patients’ cluster 2 + 3 vs. 4 + 5 + 6) and constitute an exploitable signature for molecular prediction of ICIs’ efficacy. Indeed, we show a clear segregation of early-progressors cluster 4 + 5 + 6 and vs. long responders (patients clusters 2 + 3) (**Figure 3A’**). We first focused on genes of early progressors (gene clusters 4 + 5 + 6 in **Figure 3A’**) and found that these were mainly involved in the following functions: Immune effector process/PD-1 signaling/Positive regulation of T-cell activation/Co-stimulation by the CD28 and IFN signaling (respective log p-value=-13.7, -10.2, -7.7, -10.3 and -8.5: sharing some common genes HLA-DQA1/DQB1, HLA-DRA, HLA-DRB1/DRB5), as well as regulation of IL-1 production (log p-value=-3.3: CD33, EGR1, TYROBP, AIM2, HAVCR2) and IL-18 signaling/ERBB1 downstream pathway (respective log p-value=-3.3, -2.4 respectively: BCL2L1, PTPN7, PIGT, EGR1, SLC9A1) (**Figures 3A, A’, B**, **Supplementary Table 3B**). Notably, HLA-DRB and HLA-DQ genes have been recently highlighted to be key players in different immune cells of healthy individuals as well as in whole blood samples of patients with chronic inflammatory conditions such as rheumatoid arthritis (41). Finally, we found some early-progressed patients with positive regulation of I-kappaB kinase/NF-kappaB signaling (log p-value=-3.0: CASP10, PPP5C, TRIM62) and cellular lipid catabolic process (log p-value=-4.4: LIPE, PLA2G6, SESN2, DAGLB). In contrast, when we analyzed the genes expressed more in long-responders to ICIs (gene clusters 1 + 2 + 3, **Figure 3A’**), we found the activation of pathways for negative regulation of intracellular signal transduction (log p-value=-2.2: DAG1, PER1) and focal adhesion via PI3K-Akt-mTOR-signaling (log p-value=-4.1: FGFR1, ITGA5, PFKFB3). In **Figure 3C**, we present a curation of genes that can constitute the basis for designing a panel of genes that predict response efficacy upon ICIs. In practice, RT-qPCR analysis on sorted CD4^+^T-cells can determine the propensity of patients to express genes associated with long response or early progression. In order to hint to regulatory mechanisms ruling these transcriptomic patterns, we checked transcription factors (TFs) that might regulate the expression of these identified genes. Indeed, the TF binding sites for RFXAP, RFX5, RFXANK, CIITA and ILF3 were significantly upregulated in cluster 5 including HLA-DQA1/DQB1, HLA-DRA, HLA-DRB1/DRB5 gene promoters (**Figure 3B'**). The regulation of these genes’ expression by *RFXANK*, *RFXAP*, and *RFX5* TFs has been pointed across 18 different cancer types. The *RFXANK*, *RFXAP*, and *RFX5* TFs form the RFX trimeric complex (42) which cooperates with *NLRC5* to drive the transcription of abovementioned MHC-associated genes (e.g., HLA-DQA1/DQB1, HLA-DRA, HLA-DRB1/DRB5) (43).

### 3.5 Integration of molecular signatures defining melanoma staging and ICI effects

**Figure 4:**
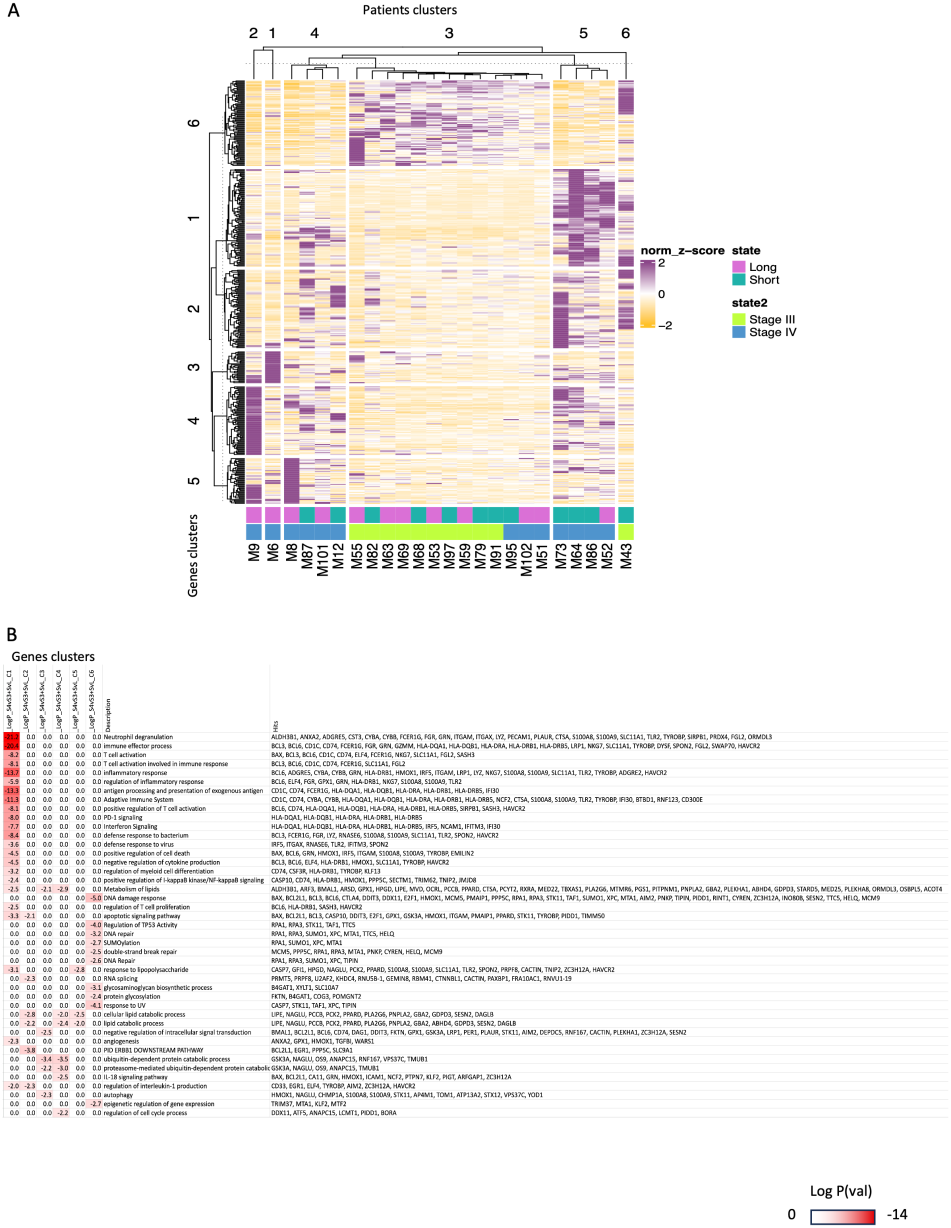
Integrated transcriptomic analysis of CD4^+^T-cells from peripheral blood prior to ICI initiation of patients with stage III and IV melanoma distinguished them as long responders and early progressors to immunotherapy. **(A)** The panel A is a comparative heatmap of selected GO terms found to be enriched in both comparisons (between patients with stage III vs. patients with stage IV melanoma of **Figure 2** and between long responders vs. early progressors to ICIs of **Figure 3**). **(B)** The panel B is k-mean clustering of patients (columns) and genes(rows) to identify group of genes that characterize better specific subsets of patients (e.g., the long responders with stage IV melanoma). Darker red color signifies most enriched GO categories and gene names contributing to the signatures are highlighted on the right.

To further take advantage of our results, we performed an integrative analysis by combining all the genes we found in **Figure 2** and **Figure 3**. In **Figure 4A** we observed a relatively robust clustering of a fraction of the diagnosed patients with stage IV patients exclusively segregated to patients’ cluster 1 + 2 + 4 + 5), while stage III patients were gathered in cluster 3 + 6. This result constitutes another exploitable signature for molecular staging of melanoma and for predicting of response to ICIs but it should be interpreted with caution. In particular, defined groups of these genes characterize long responders vs. early progressors stage IV patients (patients’ clusters 1 + 2 + 4 vs. 5 in **Figure 4A**), while the stage III patients appear to show limited segregation as far as their predictive output to ICIs (patients’ clusters 3 + 6 show both long responders and early progressors). On the other hand, looking among these clusters (for example, in clusters 4 + 3 + 5) we can detect both stage IV early progressors (SHORT) and stage IV long responders (LONG). Functional GO analyses probably reveal a limited overlap in functions demarcating stage III vs. stage IV and for the latter the genes dictating the differences between long responders vs. early progressors (**Figure 4B**, **Supplementary Table 4B**). The implication of such a GO enrichment analysis in a bigger cohort of melanoma patients could give us a clearer separation of DEGs and could allow us to find more specific differences in gene expression between early progressors (SHORT) vs. long responders (LONG) within each stage. Even though, current analysis highlights some cases of particular clinical interest. First, we note that 3 out of 4 stage IV patients with inflammatory signature prior to ICI show early progression phenotype (**Figure 4A**, gene cluster g1 and patient cluster p5). Other GO pathways that were found to be enriched in stage IV (**Figure 2**, adaptive immune system, T-cell activation, etc.) are not found in long responders (gene cluster 1 low in patients’ clusters 1 + 2 + 4) (**Figures 4A, B**). In fact, long responses under immunotherapy can be obtained based on a given transcriptomic profile especially for stage IV patients expressing genes involved with metabolism of lipids, ubiquitin-dependent protein catabolic process or IL-18 signaling pathway (cluster 1 + 2, **Figure 4A**).

## 4 Discussion

In this hypothesis-generating study, we initially examined whether the baseline immune subpopulations of peripheral blood differ among patients with locally advanced and metastatic melanoma, or among early progressors (e.g., RFS/PFS<12 months) and long responders (e.g., RFS/PFS>12 months) to ICIs; next, since CD4^+^T-cells serve as a central nexus directing the initiation and coordination of immune response, we focused on this cellular subset and explored in selected individuals with extreme clinical outcomes if there are any transcriptomic signatures on circulating CD4^+^T-cells that reflect melanoma staging and ICI responsiveness. Our findings of similar immune cellular populations in both sets of analysis (e.g., staging and ICI responsiveness) are consistent with those in patients with biliary tract cancers; when they were analyzed according to their immunotherapy response (44), but differ from those reported in other melanoma patients, in which a lower frequency of CD4^+^and CD8^+^T-cells, and a higher frequency of CD19^−^HLA-DR^+^myeloid cells at baseline was observed in responders compared to non-responders to ICIs (32). Several studies have targeted peripheral CD4^+^T-cells in human cancers in peripheral blood or in tertiary lymphoid structures (45–47). Kagamu et al. showed that NSCLC patients with decreased numbers of circulating CD4^+^T-cells and low CD62 expression(e.g., a mediator of T-cell priming and migration to secondary lymphoid tissue), developed earlier acquired anti-PD-1 resistance, in contrast to long responders characterized by increased numbers of these CD4^+^T-cells in the peripheral blood (45). In melanoma patients after anti-CTLA-4 treatment, peripheral CD4^+^T-cell clones proliferate and are enriched in corresponding tumors (47). Recently, Lucca et al. analyzed paired transcriptome and TCRαβ repertoire of circulating CD4^+^T-cells and TILs from matched samples of patients with metastatic melanoma and found that in circulating CD4^+^T-cells matching clonally expanded TILs, gene signatures of effector functions reflect those observed in the tumor (46). In parallel, activated CD4^+^memory T-cell abundance in the peripheral blood and TCR diversity at baseline were recognized as predictors of irAEs in metastatic melanoma patients (48). A notable correlation between early T-cell clonal expansion and the onset of severe irAEs was also confirmed in patients treated with ICI doublets. These studies have exemplified the usefulness of single-cell RNA-sequencing (scRNA-seq) in immunotherapy research while the paired technique of scRNA-seq with single cell T-cell receptor sequencing (sc-TCRseq) may provide further information on T-cell differentiation, specificity and activation to better understand underlying etiology and guide future strategies (49, 50).

After sampling of our study cases with extreme clinical outcomes to ICIs, DEA and GO enrichment data showed that some individuals clustered separately, independently of their staging at diagnosis, since specific biological pathways and potential marker genes are upregulated. However, it is unclear whether these transcriptomic alterations and activated pathways of adaptive immunity are reflections of the intra-tumoral plasticity to the peripheral blood or if these modifications in circulating CD4^+^T-cells actually allow melanoma to differentiate, to resist and to metastasize. The transcriptional switching from the proliferative-to-invasive phenotype of melanoma cells remains one of the main escape mechanisms of immune surveillance, mainly induced through extracellular TME signals (51). The involvement of different immune cells (52), the metabolic conditions such as oxygen and nutrient supplies (53), as well as the administered therapies can dynamically affect these outcoming phenotype (54). The Innate anti-PD-1 Resistance (IPRES) changes are mainly transcriptional and non-genomic modifications that drive the heightened mesenchymal-to-invasive transition and concurrent overexpression of genes involved in the cell adhesion, extracellular matrix remodeling, and angiogenesis (55, 56). Checking for upregulated RNA-seq only in tumor samples, Hugo et al. suggest that attenuating these biological processes that underlie IPRES may improve anti-PD-1 melanoma response (55). Herein, we argue that the intra-tumoral plasticity may be functionally depicted to the peripheral immune components (and especially in the vital CD4^+^ subpopulation) and vice versa; the changes in adaptive immunity upon TME modifications and mesenchymal-to-invasive transition, are two processing profiles of the same interplay that are happening simultaneously, with unknown priority and yet this interplay fosters too little attention.

The CD4^+^T-cell profile of metastatic melanoma patients was more enriched for inflammatory response, adaptive immunity processes, T-cell activation and lipids’ metabolism. Tumor growth is affected by abnormal adaptive immune responses such as inflammatory environment, aggressive clones, induced immunosuppression and metastatic potential (57). In preclinical models, COX-2 inflammatory activity was suggested as key determinant of immune escape in multiple cancer types (58, 59). When mice with growing tumors were treated with an ICI along with an anti-inflammatory COX-2 inhibitor (celecoxib), up to 70% of them responded to the combined treatment with many having their tumors fully eradicated, compared to <30% responded to ICI monotherapy (60). Non steroid anti-inflammatory drugs such as COX-2 inhibitors can rapidly alter the immune landscape by cutting off the cancer’s escape route and by increasing the intratumoral accumulation of effector T-cells, enhancing tumor immunogenicity and susceptibility to ICIs. Similar results were also achieved when combining ICIs with steroid anti-inflammatory drugs - a surprising finding, as steroids are widely considered to suppress the immune system. In fact, concurrent targeting of tumor inflammatory profile with a COX-2 inhibitor together with an ICI significantly improved 6-month PFS and ORR in patients with metastatic melanoma and NSCLC compared with ICI alone (61). In patients with dMMR/MSI-high locally advanced rectal cancer, the addition of celecoxib to neoadjuvant toripalimab offered a pCR of 88% (62). Even in patients with pMMR/MSS disease, NICHE trial observed a pathological response rate of 30% (9 of 30) after a single dose of ipilimumab and two doses of neoadjuvant nivolumab with 4 of 9 responders receiving celecoxib (63). Combinations of celecoxib with ICIs are also being tested in other cancer types (e.g., LION trial). Following our transcriptomic-based hypothesis, directly metastatic cases or non-responders to ICIs could be those that overexpressed inflammatory genes. Our stage III melanoma patients don’t show an enhanced inflammatory profile, and for this reason may be able to keep an active and stable response while some patients with metastatic spread seem to have an already solicited immune system (**Figure 3A**; patients’ clusters 5 and 6) that probably could not keep immune surveillance for a long duration after triggering by anti-PD-1/anti-PD-L1 ICIs. Indeed, the pre-ICIs inflammatory response pathway is significantly upregulated to stage IV and early progressors subgroups (**Figure 2A’** - gene cluster 3 high in patient cluster 5, and in 3A ‘gene cluster 5 high in patient cluster 5 + 6), and we even find that 3 out of 4 stage IV patients with inflammatory signature prior to ICI show early progression phenotype (**Figure 4A**, gene cluster g1 and patient cluster p5). The biomarker analysis of Checkmate76K phase III trial announced that the lower CRP levels at the serum were associated with longer RFS with adjuvant nivolumab in stage IIB/IIC melanoma (64). In a simplified interpretation, patients with stage III over-expressing genes on DNA repair pathways (e.g. DNA repair, response to UV, DSB repair, regulation of cell cycle, DNA damage response, chromatin organization) (**Figure 2A’** and GO panel, gene clusters c5 and 6 and patient cluster 1 + 3) and in parallel, under-expressing inflammatory genes (e.g. involved in inflammatory response and adaptive immunity: expressed in **Figure 2A’** - gene cluster 3, **Figure 4A’** gene cluster 3) seem to have greater possibility to not experience a metastasis (11 stage III patients out of 14 in patient cluster 1 + 3) or to avoid an early relapse of their disease under adjuvant immunotherapy especially for stage IV patients (2 stage IV patients out of 3 with long response in patient cluster 3 **Figure 4A’**) as stage III patients show half-half chance to respond (**Figure 4A’** patient cluster 3).

In early progressors, highly upregulated transcripts were recognized in immune effector processing, PD-1 signaling, positive regulation of T-cell activation, co-stimulatory molecules of CD28 family and IFN signaling. The interaction of TCR with an MHC-presented antigen is followed by the concurrent binding of a CD28 costimulatory molecule, providing a second signal alongside TCR ligation, more complex in both binding pattern and biological effects. The subsequent downstream propagates with various effector enzymes, such as kinases, phosphatases, and phospholipases (65, 66). Gathering of mutations in the phosphorylation of CD3 molecules may disrupt their ability to bind to the TCR molecules forming a functional complex or may impair its transduction ability (67). In continuation, the receptors of CD28 family (e.g., CD28, CTLA-4, PD-1, TIGIT, ICOS, and BTLA) may have diverse effects on T-cell functions, including membrane raft trapping at the immunological synapse, transcriptional changes, downstream post-translational modifications, and actin cytoskeletal remodeling, leading to many intracellular biochemical events such as survival and proliferation signals, induction of IL-2, activation of telomerase, stabilization of mRNA for several cytokines, increased glucose metabolism, and enhanced T-cell migration and homing (68–70). For instance, CD28 (activating) and CTLA-4 (inhibitory) are highly homologous and compete for the same ligands (CD80 and CD86) and regulate immune response by providing opposing effects (68, 69). On the other hand, blocking of PD-1/PD-L1 axis has been proven to restore the effector function of T-cells and improve T-cell priming, with significant survival benefit for treated patients (71). However, as described above, it is known from chronic infections, that continuous stimulation of PD-1 expression on antigen specific T-cells is also positively associated with T-cell exhaustion (72). The timing and the duration of expression of the identified MHC-associated genes (e.g., HLA-DQA1/DQB1, HLA-DRA, HLA-DRB1/DRB5) and concurrently of the targetable checkpoint receptors (e.g., CTLA-4, PD-1, LAG-3, TIGIT, etc.) looks to be more crucial than their baseline levels before the initiation of ICIs (73). In the early progressors, the RFX-mediated upregulation of identified HLA genes and MHC-associated pathways may confirm a pre-existing inflammatory condition and an already exhausted immune system, unable to inhibit melanoma spread. At this point, it is worth to be noticed that the upregulation of some genes in a cellular pathway (for instance, the PD-1 signaling) does not mean that the related targetable checkpoint molecules are definitely overexpressed). In our sub-cohort of early progressors, the over-expressed BCL6 in many GO pathways is a major negative regulator of PD-1 signaling that directly binds to the promoter region of PD-L1 and to the intron 2 of PD-L2 to suppress their transcription and in addition, represses the expression of STAT1/STAT3/IRF1 and indirectly inhibits the transcription of PD-1 ligands. This could justify the similar MFI expression of PD-1 on circulating CD4^+^T-cells in contrast to the different levels of enrichment of PD-1 signaling in the two sets of analysis. Recently published complementary data on the TME of HNSCC, NSCLC, and melanoma: i) discriminated five different implicated T-cell subtypes (e.g., naïve, activated, exhausted, effector memory, and central memory T-cells), ii) recognized that CD39^+^and PD-1^+^surface markers could accurately predict response or exhaustion, and iii) identified specific T-cell subpopulations or specific T-cell gene profiling associated with anti-PD-1 response (74). Both *in vitro* and in-patient findings agreed that many traditional markers were correlated with, but not specific to, T-cell exhaustion (74). Antigen-specific T-cells represent a heterogenous subpopulation whereby the increased expression of co-inhibitory receptors additional to PD-1 (e.g., TIGIT, TIM3, and LAG3) and some unique transcriptomes contribute to their overall dysfunctional status (73, 75, 76).

Unplugging the exclusiveness of PD-1 with the ICI-response, Beasley et al. found in four melanoma patients with durable response to anti-PD-1 treatment (median PFS=2.3 years) higher pretreatment tumor CD8^+^T-cell infiltrates and significantly higher effector memory (CD8^+^/CCR7^-^/CD45RA^-^) but lower CD8^+^PD-1^+^and CD4^+^PD-1^+^cells compared to eight patients with a median PFS 1.6 months (77). The expression of PD-L1 in melanoma cells is mainly regulated by IFN-γ signaling through the JAK1/2-STAT2/3-IRF1 axis, whereas PD-L2 is regulated by IFN-β and IFN-γ through both IRF1 and STAT3, which bind directly to PD-L2 promoters and promote immunosuppression (78).The upregulation of IFN-γ signaling with HLA-DQA1, HLA-DQB1, HLA-DRA, HLA-DRB1, HLA-DRB5, NCAM1 genes popping up was also observed in our analysis of early progressors vs. long responders in gene cluster 5 as shown in **Figure 3A’**. Chronic triggering of IFN-γ signaling is associated with the expression of other checkpoint ligands via STAT1-regulated epigenetic mechanisms (79) and with the induction of IDO expression, which recruits immunosuppressive T_regs_ in the TME (80, 81). After the establishment of an inflammatory TME through IFN networks, tumor cells gain STAT3 activity through immune-derived IL-10, IL-6, NF-κB, or Bcl2, which promote proliferation, antiapoptotic signals, and angiogenesis and additionally, these secreted factors drive expansion of MDSCs and T_regs_, which, together with macrophages and DCs, produce immunosuppressive TGF-β and IL-10 cytokines and also express other immunoregulatory molecules, including arginase, inducible NO synthase, and IDO (82, 83). In the same direction, Benci et al. showed that IFN signaling in cancer cells and immune cells oppose each other to establish a regulatory relationship that limits both adaptive and innate immunity. While inhibiting tumor IFN-γ signaling decreases IFN-stimulated genes (ISGs) in cancer cells, it increases ISGs in immune cells by enhancing IFN-γ mediated T-cell exhaustion. In preclinical models, type I IFN receptor or JAK/STAT inhibition suppresses melanoma-PD-1 expression and disrupts ICI efficacy (84). In the neoadjuvant setting of stage III melanoma, preliminary data connect initially increased IFN signature with tumor inflammation and immune sensitivity (85, 86). Baseline high IFN signature is associated with similar responses and EFS for ICI-monotherapies and ICI-doublets while only the nivolumab/ipilimumab combination can restore the immunotherapy response in melanoma patients with low IFN signature (85, 86). In addition, patients with IFN algorithm low who remain low on treatment do not respond even in nivolumab/ipilimumab combination (87).Together, these findings indicate the existence of a molecular context linking melanoma dedifferentiation and IFN-γ-signaling and the perturbation of this balance by the IFN-regulated messages may lead to immune evasion and may adjust ICI responsiveness, independent of tumor mutational burden (88).

Last, we provide evidence that the lipids metabolism may be implicated in melanoma metastatic status and its response to immunotherapy (**Figures 2B**, **3B**, **4B**). In general, cellular metabolism controls T-cell differentiation, survival, and effector functions in many types of cancer. Recently, Liu et al. observed that both malignant cells and T_regs_ can alter the lipid metabolism via elevated expression of group IVA phospholipase A_2_, and can induce T-cell senescence (89). Senescent T-cells have active glucose metabolism but exhibit unbalanced lipid metabolism, which, in turn, results in accumulation of lipid droplets in T-cells. In responder T-cells during senescence, MAPK signaling and STAT signaling coordinately control lipid metabolism and activity of group IVA phospholipase A_2_. Inhibition of group IVA phospholipase A_2_ reprogrammed effector T-cell lipid metabolism, prevented T-cell senescence *in vitro*, and enhanced antitumor immunity and immunotherapy efficacy in melanoma mouse models. In agreement with our findings, the authors concluded that there are mechanistic links between the regulation of lipid metabolism and the T-cell senescence, providing further insights for tumor immunotherapy (89).

The exploratory nature of our study has several critical gaps and limitations. Our sample of consecutive melanoma subjects used for peripheral immunophenotyping represents a small and heterogeneous population that could not disclose meaningful differences among the two sets of analysis. Therefore, we exclusively focused on the RNAseq of peripheral CD4^+^T-cells of extreme responders and non-responders. This selective sampling, although unrepresentative of the full spectrum of patient responses, was performed to develop an initial idea and hypothesis and to identify initial patterns for further testing and definitely not for extracting strong conclusions. Further validation and replication of these results into other larger cohorts are required to establish broader applicability. Moreover, the study skips over subjects with stages I and II melanoma that could work as controls to verify if our findings hold up across earlier stages, supporting a gradual escalation of GO expression and making these results more universally applicable. Our initial cohorts were equivalent regarding age and gender (**Table 1**) but following the different therapeutic options in each stage, the impact of treatment selection is not correctly estimated on the extreme responders and non-responders. Following the investigational setting of our study, there are also imbalances in the marker antibodies used for immunophenotyping; markers to identify three monocyte populations were included whereas our T-cell subpopulations are not comprehensively characterized; for example, by adding the CD25-antibody for activated T_regs_, or by adding the CD3-antibody for the T-cell origin in the flow cytometry panel to avoid overlap of spectra and minimize compensation and spillover. Another limitation concerns the different detection of CD4^+^cell subpopulation with fluorochrome for flow cytometry and with a clonal CD4-antibody for CD4^+^cells’ isolation and RNA-seq, inserting the bias of the higher affinity of antibody-linkage. As with the majority of studies on blood-borne biomarkers, we examined a single immune cell subtype in isolation, rather than attempting to integrate the genetic information from the multiple components of peripheral immunity. Given the broader lack of differences among peripheral immune subsets, the additional analysis narrowing to CD4^+^T-cells in a predetermined population further limit the generabilityof our data rather than objectively offering genuine insight. For instance, the study overlooks CD8^+^T-cells, which are critical in antitumor immunity and we could have a more comprehensive picture of the peripheral immune landscape by including CD8^+^ and other immune cell populations. It would be also insightful to compare the peripheral blood immune subsets with those identified directly in the tumor. This could shed light on whether these circulating cells truly reflect the TME.

Our study has not identified a specific panel of genes or transcriptomic signatures that could be implemented in clinical practice, but mainly outlined the potential utility of DEGs in detecting intracellular biomarkers and pathways in the peripheral CD4^+^T-cells that may characterize a totally different, individualized, behavior of patient’ adaptive immunity. RNA-seq is reasonably proposed here as a possible diagnostic tool but still more work needs to be done and many challenges like scalability and cost to be improved, to unveil the effect of “dark” melanoma epigenomic background and to find practical implementations of any transcriptomic signature. Adding on the already known biomarkers of LDH and of IFN-γ signature, this study provides an initial novel idea based on more time-consuming and expensive methods, compared to current existing ones, focusing mainly on better understanding of the various CD4^+^T-cell mediated systemic reactions, and on recognizing distinct extra-tumoral phenotypes. Deeper knowledge of the behavior of CD4^+^T-cells holds vast potential in guiding future cellular anti-melanoma treatments. As noticed previously, this is an exploratory study including bioinformatic analysis from a small cohort and cannot support any stronger conclusion without further validation data. Additional transcriptomic information from replicated melanoma cohorts and functional RNA-seq analyses including all immune cellular components is required to illuminate more the complex network of immune-mediated interactions in the peripheral blood of melanoma patients.

## Data Availability

The data presented in the study are deposited in the Gene Expression Omnibus (GEO) repository, accession number GSE292798.

## Supplementary material

Supplementary Figure 1
**(A)**. Gating strategy for identifying T-cell immune populations by using flow cytometry (CD4^+^, CD8^+^, CD4^+^FOXP3^+^, CD4^+^FOXP3^-^, CD4^+^FOXP3^-^Tbet^+^ and CD4^+^FOXP3^-^RORγt^+^). **(B)**. Gating strategy for identifying myeloid cell populations by using flow cytometry (HLA-DR^+^, HLA-DR^+^CD14^+^CD16^-^, HLA-DR^+^CD14^+^CD16^+^ and HLA-DR^+^CD14^-^CD16^+^). **(C)**. Gating strategy for identifying myeloid immune cell populations by using flow cytometry (HLA-DR^-^, HLA-DR^-^CD15^+^CD33^+^).

Supplementary Table 1 Summary of antibodies’ information used in Flow Cytometry.

Supplementary Table 2 Indexes and number of reads obtained. S4L means a patient with stage 4 melanoma and long PFS under ICIs/long immunotherapy responder while S3S means a patient with stage III melanoma and short RFS under ICIs experiencing an early immunotherapy relapse. RFS=Relapse Free Survival; PFS=Progression Free Survival.

Supplementary Table 2 . GO enrichment analysis using the cluster of genes found in **Figure 2A’** for stage III and stage IV. Darker red color signifies most enriched (decreasing -Log Pval) GO categories and gene names contributing to the signatures are highlighted on the right.

Supplementary Table 3 . GO enrichment analysis using the clusters of genes found in **Figure 3A’** for early progressors and long responders. Darker red color signifies most enriched GO categories and gene names contributing to the signatures are highlighted on the right.

Supplementary Table 4 . GO enrichment analysis using the combined list of DEGs found in **Figure 4A**. Darker red color signifies most enriched GO categories and gene names contributing to the signatures are highlighted on the right.
