# Supplementary figures and images for: Transcriptomic signatures in peripheral CD4+T-lymphocytes may reflect melanoma staging and immunotherapy responsiveness prior to ICI initiation
Source: Front Immunol. 2025 Mar 28;16:1529707. doi: 10.3389/fimmu.2025.1529707 (PMC11986426; doi:10.3389/fimmu.2025.1529707)

## Slide 1
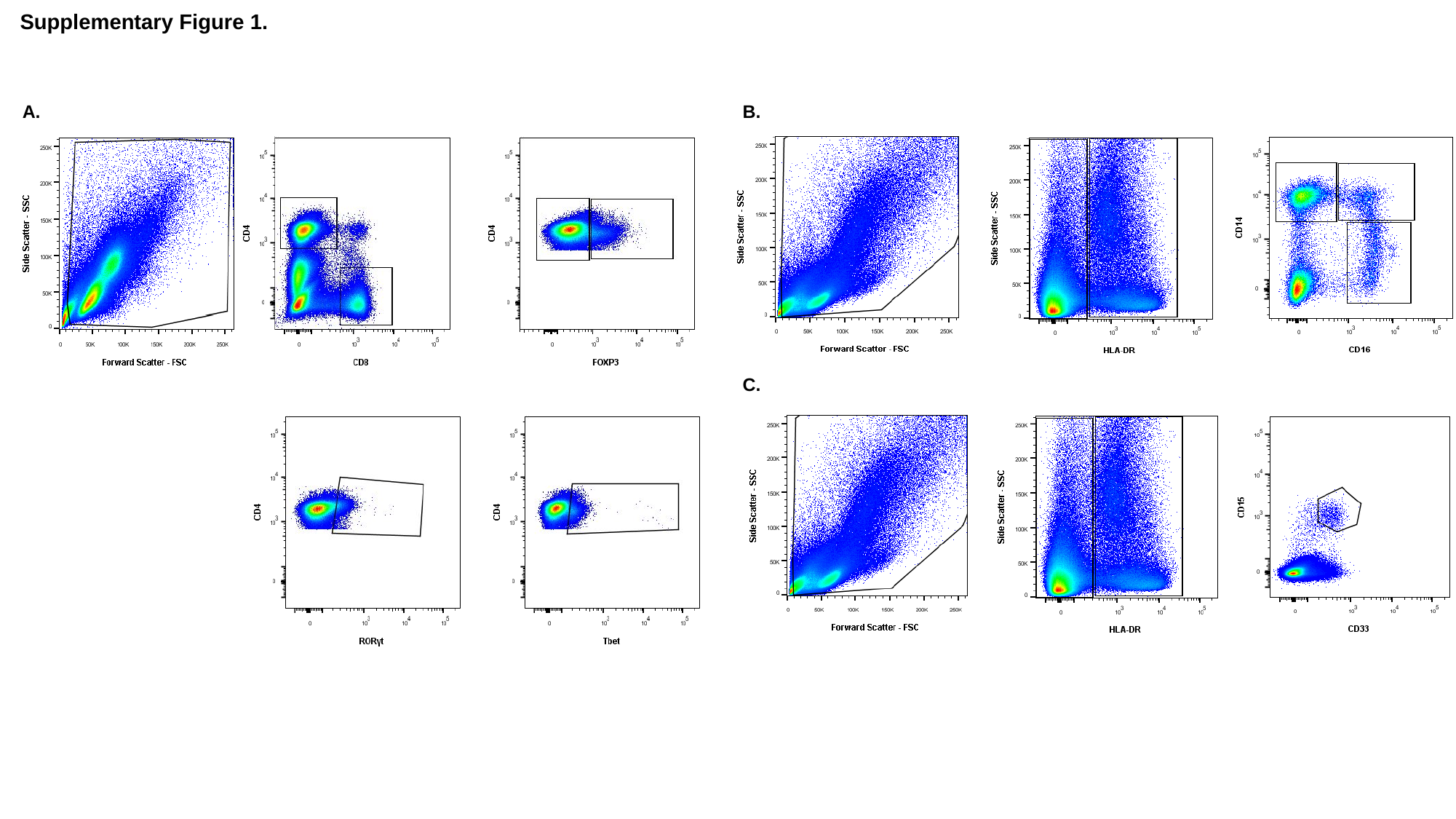

Supplementary Figure 1.
A.
B.
C.

Supplement: Supplementary Figure 1 — (A). Gating strategy for identifying T-cell immune populations by using flow cytometry (CD4+, CD8+, CD4+FOXP3+, CD4+FOXP3-, CD4+FOXP3-Tbet+ and CD4+FOXP3-RORγt+). (B). Gating strategy for identifying myeloid cell populations by using flow cytometry (HLA-DR+, HLA-DR+CD14+CD16-, HLA-DR+CD14+CD16+ and HLA-DR+CD14-CD16+). (C). Gating strategy for identifying myeloid immune cell populations by using flow cytometry (HLA-DR-, HLA-DR-CD15+CD33+). [file Presentation1.pptx]
