# Supplementary material for: Transcriptomic signatures in peripheral CD4+T-lymphocytes may reflect melanoma staging and immunotherapy responsiveness prior to ICI initiation
Source: Front Immunol. 2025 Mar 28;16:1529707. doi: 10.3389/fimmu.2025.1529707 (PMC11986426; doi:10.3389/fimmu.2025.1529707)
Supplement: Supplementary Table 1 — Summary of antibodies’ information used in Flow Cytometry. [file Presentation2.pptx]

## Slide 1
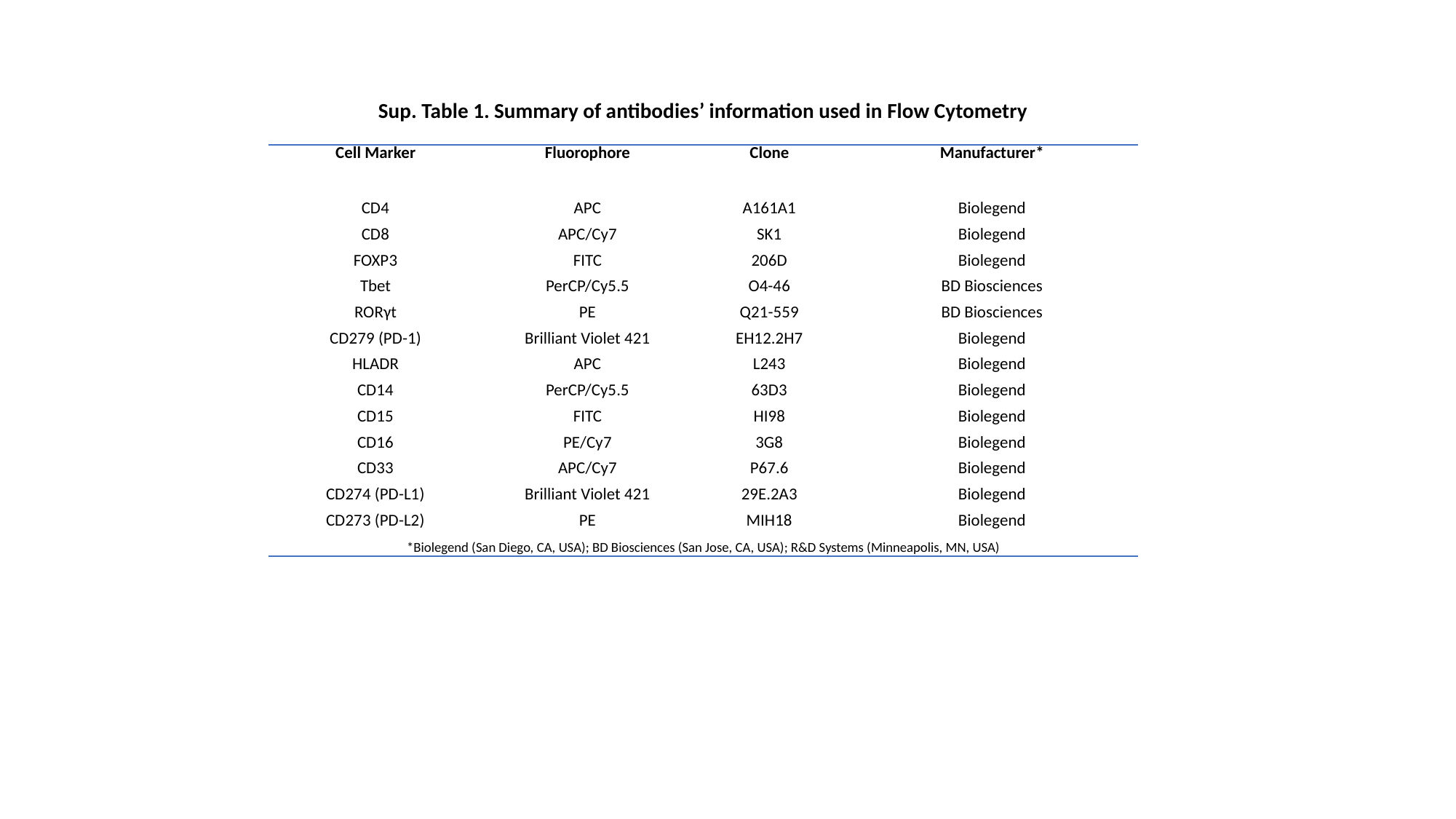

Sup. Table 1. Summary of antibodies’ information used in Flow Cytometry
| Cell Marker | Fluorophore | Clone | Manufacturer\* |
| --- | --- | --- | --- |
| CD4 | APC | A161A1 | Biolegend |
| CD8 | APC/Cy7 | SK1 | Biolegend |
| FOXP3 | FITC | 206D | Biolegend |
| Tbet | PerCP/Cy5.5 | O4-46 | BD Biosciences |
| RORγt | PE | Q21-559 | BD Biosciences |
| CD279 (PD-1) | Brilliant Violet 421 | EH12.2H7 | Biolegend |
| HLADR | APC | L243 | Biolegend |
| CD14 | PerCP/Cy5.5 | 63D3 | Biolegend |
| CD15 | FITC | HI98 | Biolegend |
| CD16 | PE/Cy7 | 3G8 | Biolegend |
| CD33 | APC/Cy7 | P67.6 | Biolegend |
| CD274 (PD-L1) | Brilliant Violet 421 | 29E.2A3 | Biolegend |
| CD273 (PD-L2) | PE | MIH18 | Biolegend |
| \*Biolegend (San Diego, CA, USA); BD Biosciences (San Jose, CA, USA); R&D Systems (Minneapolis, MN, USA) | | | |
